# Towards Celiac-safe foods: Decreasing the affinity of transglutaminase 2 for gliadin by addition of ascorbyl palmitate and ZnCl_2_ as detoxifiers

**DOI:** 10.1038/s41598-017-00174-z

**Published:** 2017-03-06

**Authors:** N. Engstrom, P. Saenz-Méndez, J. Scheers, N. Scheers

**Affiliations:** 1 Chalmers University of Technology, Department of Biology and Biological Engineering, The division of Food and Nutrition Science, 412 96 Gothenburg, Sweden; 2 University of Gothenburg, Department of Chemistry and Molecular Biology, 405 30 Gothenburg, Sweden; 3 UdelaR, Facultad de Química, Computational Chemistry and Biology Group, 11800 Montevideo, Uruguay; 4 Chalmers University of Technology, Department of Physics, 412 96 Gothenburg, Sweden

## Abstract

Initiation of celiac disease is triggered in the gastrointestinal tract by transglutaminase 2 (TG2) assisted deamidation of gluten peptides. Deamidation is a side-reaction to transamidation and occurs if primary amines are absent. In contrast to deamidation, transamidation does not trigger an immune response. The aim of the study was to identify a suitable food additive that interacts with TG2 binding motives in gluten-derived peptides to prevent deamidation/transamidation. Homology modelling of α2-gliadin and computational screening of compounds for their binding affinity to a common TG2 binding motive (P)QLP were done by using computational approaches followed by experimental testing of TG2 activity. A database containing 1174 potential food grade ligands was screened against the model of α2-gliadin (27 out of 33 aa). Out of the five best ligands, ascorbyl palmitate, was observed to decrease TG2 transamidation of gliadin by 82% ± 2%. To completely silence the transamidation, we added zinc chloride (ZnCl_2_), and thereby reached a 99% ± 1% inhibition of TG2 activity. In addition, we conducted a pilot experiment in which ascorbyl palmitate was observed to decrease TG2 deamidation of gliadin completely. We propose ascorbyl palmitate in combination with ZnCl_2_ with the future perspective to become an additive in celiac-safe foods.

## Introduction

### Celiac disease

Celiac disease, or gluten intolerance, is an autoimmune inflammatory disease mainly affecting the mucosal lining of the gastrointestinal tract. The classical symptoms including pain, diarrhoea, and malabsorption, arise when genetically pre-disposed individuals ingest foods containing gluten. Gluten is a heterogeneous group of proteins high in the amino acids glutamine and prolamine, which are present in wheat. In addition, there are similar proteins in barley and rye (hordeins and secalins), which also may provoke an immune response in celiacs. The incidence of celiac disease is increasing and varies much depending on region e.g. in the US the number of diagnosed and undiagnosed cases in 2012 were estimated to be 0.75% of the population^1^ compared to Northern Europe where the prevalence of celiac disease is estimated to 1–2%^2, 3^. There may be several causes for the regional difference in prevalence such as high intake of cereals, unbalanced diet, and higher incidence of infections.

### Genetic susceptibility and the immune response

Individuals that develop celiac disease carry one or two specific HLA alleles in their genome; HLA DQ2 and/or HLA DQ8. These alleles code for specific major histocompatibility complex molecules (MHCs), which are surface receptors on antigen-presenting cells belonging to the immune system. The HLA DQ2 and HLA DQ8 proteins have high affinity for deamidated, negatively charged gluten-derived peptides. When fragments of the gluten-derived peptides are presented to T lymphocytes; T-cells (T-helpers, type 1), a complex is formed between the surface receptors and the antigen, which initiates an inflammatory response with the production of cytokines and recruitment of antibody-producing B lymphocytes (B-cells). The primary cytokine produced is interferon γ (IFN-γ), which is also the cytokine that maintains the inflammation in the celiac intestine. About 20–30% of the world population carry the HLA DQ2/DQ8 alleles and thereby the susceptibility to develop celiac disease. However, only about 3% of these with the pre-disposition develop the condition, indicating that there are other factors important for the initiation.

### Initiation of the immune response to deamidated gliadin peptides

Stimulation of an immune response by gluten starts with the processing of gluten by transglutaminase 2 (TG2; E.C. 2.3.2.13) in the intestinal wall. TG2 is expressed in enterocytes of the upper parts of the villi and in fibroblasts along the intestine^4^. The reaction is initiated when partly digested gluten peptides encounter TG2, which forms a thioester bond with specific glutamine (Gln) residues in an acylation reaction releasing ammonia (NH_3_) as a product. If a primary amine is present, the glutamine residues will be transamidated. If no primary amine is available for the crosslinking, the glutamine residues will instead be deamidated, forming the negatively charged residue glutamic acid (Glu). The last step is hydrolysis of the thioester bond, leaving a negatively charged gluten-derived peptide. The hydrolysis is the rate-limiting step of the reaction, which results in a build-up of the thioester-deamidated gluten peptide in the mucosa and thereby increases the probability of T-cell activation.

### Affinity of TG2 to gluten proteins

Transglutaminases are intracellular as well as extracellular enzymes which function is to cross-link proteins by transamidation of primary amines. This family of enzymes is important in the cross-linking of collagen as well as stabilizing fibrin clots during wound healing. TG2, which is the transglutaminase subtype present in the intestinal wall, catalyses either the transamidation or deamidation reaction described in the former section. The initial acylation reaction takes place at the carbonyl carbon of glutamine residues, but specific 3-aminoacid motifs on the gluten peptide are required for being a good substrate for TG2. In gluten proteins, especially the gliadin fraction, the content of glutamine and proline is particularly high. However, several other gluten peptides have been reported to stimulate a T-cell response. The presence of gluten motifs is not sufficient for initiating TG2 enzymatic activity. TG2 is dependent on Ca^2+^, which activates the enzyme by causing a conformational change, revealing the gluten-motif binding site^5^. While Ca^2+^ is required for the enzyme to stay in an open conformation, Zn^2+^ inhibits the activation by competing with Ca^2+^ for the binding site^6^. Thus, the intestinal Ca^2+^ and Zn^2+^ balance is important for the affinity of TG2 to gluten suggesting that an acquired intestinal zinc deficiency, either through dietary restriction or by infection, may initiate celiac disease.

In this work we aimed to take advantage of the molecular mechanisms for the initiation of celiac disease by identifying a compound to be used as a potential flour additive that interacts with TG2 binding motives in gluten-derived peptides to inhibit the recognition by TG2 and thus prevent its binding and thereby decrease the formation of the thioester-deamidated gluten peptide that is associated with T-cell activation in celiacs. In addition, we added ZnCl_2_ to the system in order to prevent the conformational change, the pre-requisite for TG2 trans- or deamidation activity. In our studies we used gliadin and also α2-gliadin, a proteolytically stable fragment of α-gliadin, expected to be present after gastrointestinal digestion.

## Results

### Homology model of α2-gliadin

From BLAST search with solved PDB structures^7^, we could not find any available template. However, two articles associated with the deposition of related PDB structures were found^8, 9^. Kim *et al.* reported the X-ray structure of the soluble domain of HLA-DQ2 bound to the deamidated gluten epitope alpha-I-gliadin, LQPFPQPELPY (1S9V), while Petersen *et al.* described the HLA-DQ2 complexes with two epitopes from wheat gliadin, namely QPFPQPELPYP and PQPELPYPQP. These peptides obtained after deamidation of Q to E catalysed by the enzyme transglutaminase 2 (TG2) were thus used as templates to build a homology model of alpha-2-gliadin (33-mer).

**Figure 1:**
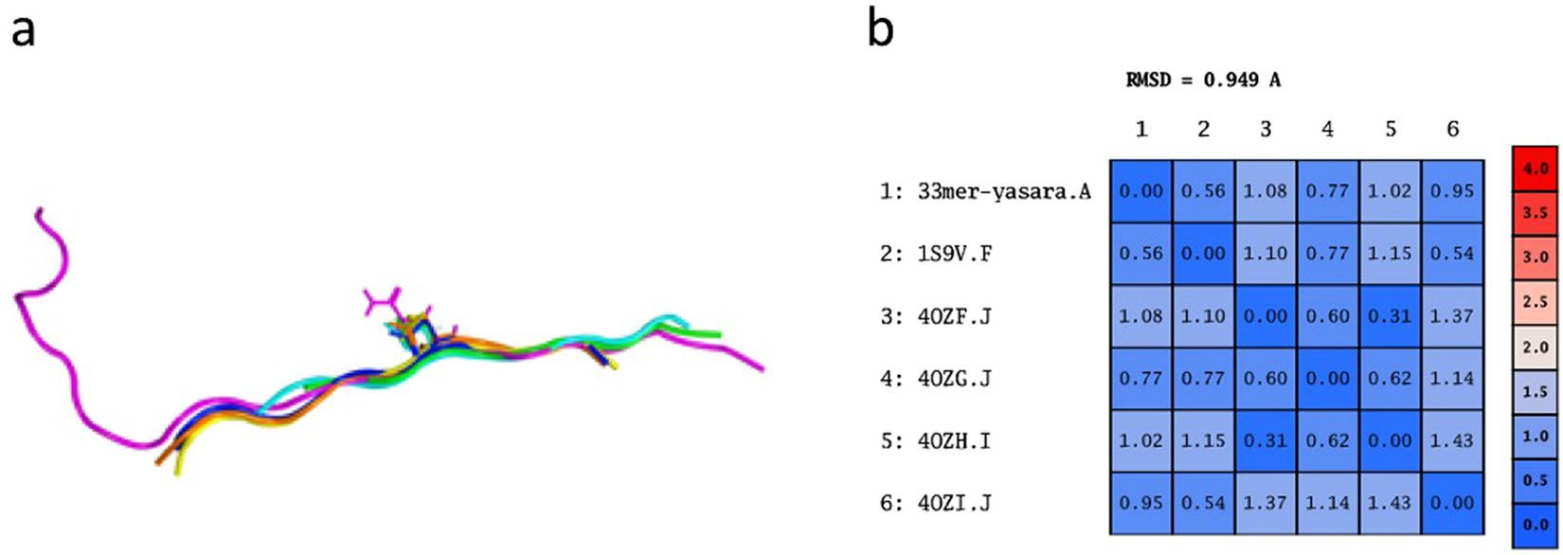
Homology modelling of α2-gliadin*.* (**a**) Superposition of the 3D structures of templates (1S9V in green, 4OZF in blue, 4OZG in yellow, 4OZH in orange, and 4OZI in cyan) and the homology model (magenta). (**b**) RMSD matrix values of the positions of the Cα atoms for each pair of peptides.

Five models were built from these templates, and further refinement was performed combining the best parts of different models in YASARA, aiming to increase the accuracy of the final model. The resulting hybrid model was then subjected to a simulated annealing minimization obtaining the highest quality model according to the overall z-score (−0.231 compared to −0.348, −0.635, −0.734, −0.787 and −1.176 for model 1 to 5, respectively). 27 out of 33 residues were modelled, skipping the C-terminal residues since YASARA does not perform ab initio structure prediction, appending a very long tail would reduce the accuracy of the final model. PROCHECK analysis of the current model shows that no residues were found in disallowed regions, 80% in most favoured regions and 20% in additional allowed regions. These results show that the model presents a good percentage of residues in the most favoured and additionally allowed regions, indicating that the backbone dihedral angles were sufficiently accurate. The backbone superposition of the 3D structures is shown in Fig. 1a. As evidenced by the RMSD calculations after alignment and superposition of the templates and the model (Fig. 1b), it is clear that the homology model superposes well with all templates, indicating high structural template-model similarity and no significant structural drift of the model from the template.

### Docking of molecules to the binding motif (P)QLP in the α2-gliadin model

**Figure 2:**
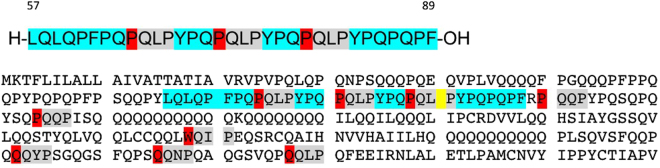
Gliadin (33 aa) and the entire α-gliadin sequence (290 amino acids). Source: Uniprot; Q1WA39. Blue high-light: α2-gliadin, “the 33-mer”. Grey highlight: TG2 binding motives. Red: The amino acid flanking the glutamine residue to which TG2 binds during the first nucleophilic attack.

**Figure 3:**
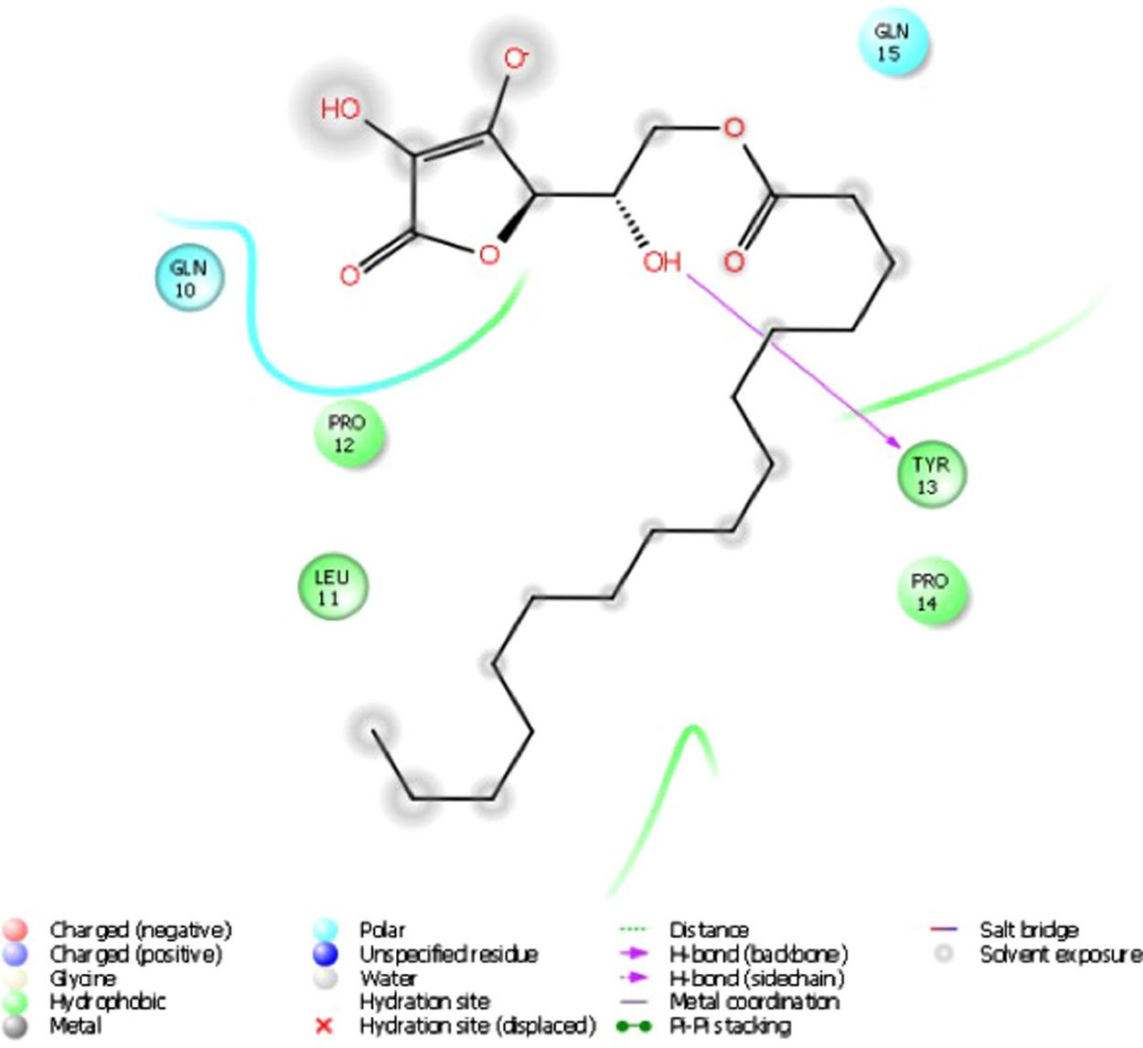
Ligand interactions between the α2-gliadin model and ascorbyl palmitate. 2D presentation.

From virtual docking studies of the 108 GRAS approved ligands (1174 entries) to the 3-fold recurrent motif (P)QLP in the α2-gliadin model, we report the binding energies of the five best ligands together with twelve other compounds (also GRAS) that we experimentally evaluated previously (Table 1). The choice of the best ligands was based on properties such as binding energy, sensory properties, and acceptable daily intake quantities. Only two ligands ranked better than the reported ones, Mg stearate and PEG mono stearate. Both were excluded due the very low solubility of Mg stearate and the common use of PEG mono stearate in personal products (shampoo, conditioners, and lotion), which will likely affect consumer acceptance. The nature of the interactions is also important e.g. Mg stearate interacts with residues QPFPQPQLP which is only present once in α2-gliadin and will therefore not cover the 2 additional PQLP motifs in the fragment (Fig. 2). In comparison, ascorbyl palmitate, ranked as no 1 in Table 1, interacts with the residues QLPYPQ (Fig. 3) and can thereby theoretically, not taking steric interactions into account, interact with all 3 motifs in α2-gliadin.

**Table 1:** Binding energies of selected ligands screened against the motive (P)QLP in the homology model of α2-gliadin.

Rank	Entry name	MMGBSA dG Bind
1	Ascorbyl palmitate	−52,790
2	Taurocholic acid	−51,688
3	Ergocalciferol	−48,361
4	Dextran	−41,784
5	Na stearate	−37,831
6	Glutathione (ox)	−35,126
7	Glyceryl diacetate	−27,114
8	Carnitine (L)	−25,935
9	Glutathione (red)	−24,207
10	Adipic acid	−20,950
11	Carnosine (L)	−20,152
12	Glyceryl triacetate	−18,783
13	Lysine (L)	−17,967
14	Cysteine (L)	−13,738
15	Lactate	−11,578
16	Taurine	−11,564
17	Succinic acid	−10,330
18	Tartaric acid	−9,210

### Ascorbyl palmitate inhibits TG2 transamidation and deamidation of gliadin

**Figure 4:**
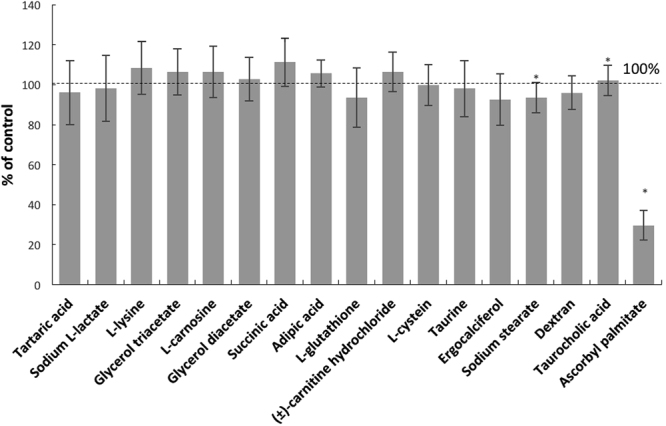
TG2-mediated transamidation of wheat gliadin (to biotin) in the presence of selected ligands at 15 µM (reported in Table 1) shown as percentage of gliadin control. Data are presented as means ± SD (n = 3). The asterisk indicates a significant change (p < 0.05) from control. Only ascorbyl palmitate was able to reduce the transamidation to a major extent.

**Figure 5:**
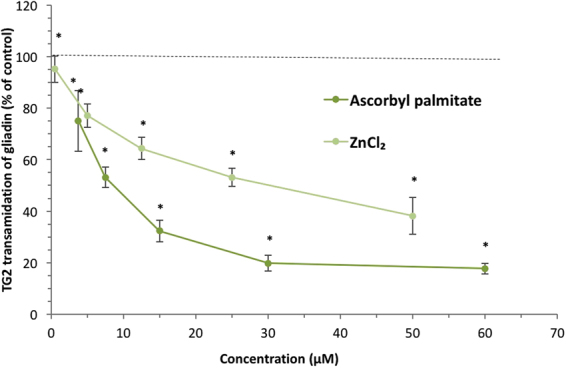
Dose-response experiments (5–60 µM) of TG2-mediated transamidation of wheat gliadin biotin (to biotin) in the presence of ascorbyl palmitate and ZnCl_2_ separately. Data are presented as means ± SD (n = 3). The asterisk indicates a significant change compared to control (p < 0.05).

**Figure 6:**
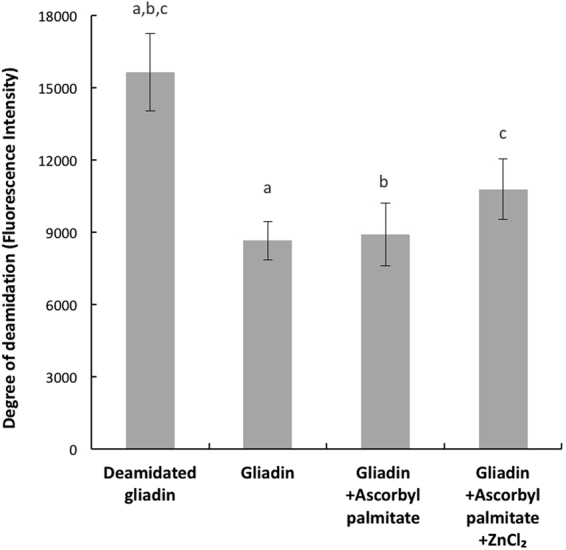
The degree of gliadin deamidation was measured by an anti-body based ELISA. A pilot experiment was conducted to confirm the inhibitory effect of ascorbyl palmitate on TG2 activity. There was no significant difference between TG2 processed ascorbyl palmitate treated gliadin and the unprocessed gliadin control (p = 0.75). No effect of ZnCl_2_ could be observed since TG2 activity was already silenced. The letters a, b, c indicate significant differences between deamidated and non-deamidated gliadin.

The virtual screening was done with the gliadin fragment α2-gliadin (33 aa) while the experimental evaluation was done with gliadin (290 aa; primary sequence and binding motifs: Fig. 2). α2-gliadin with its 3 QLP motifs is considered as the most immunogenic part of gliadin since it is stable after gastrointestinal digestion. However, there are six additional potential TG2 binding sites on the major part of α gliadin that may or may not be available after digestion. All seventeen ligands reported in Table 1, were tested for their ability to prevent transamidation of gliadin with a primary amine (here biotin) catalysed by TG2. Ascorbyl palmitate was the only ligand that sufficiently inhibited transamidation of gliadin at 15 µM, Fig. 4. The inhibition was extensive (70%) but could be further increased to 82% at 30 and 60 µM (no significant difference between 30 and 60 µM) as observed in dose-response experiments, Fig. 5. Transamidation or deamidation will take place dependent on the availability of primary amines, we used biotin (in excess) as primary amines to estimate TG2 transamidation activity. The steps before both deamidation and transamidation reactions are the same (the binding of the enzyme to gln-x-pro) and both reaction types will be prevented if the enzyme cannot bind. The options were therefore to drive the reaction fully towards transamidation or to drive it fully towards the deamidation reaction. We confirmed the effect of ascorbyl palmitate on TG2 activity by providing conditions where deamidation was favoured before transamidation and observed a complete decrease in TG2 deamidation of gliadin (no significant difference to the gliadin control), Fig. 6.

### The combination of ascorbyl palmitate and ZnCl_2_ for the complete inhibition of TG2 activity

**Figure 7:**
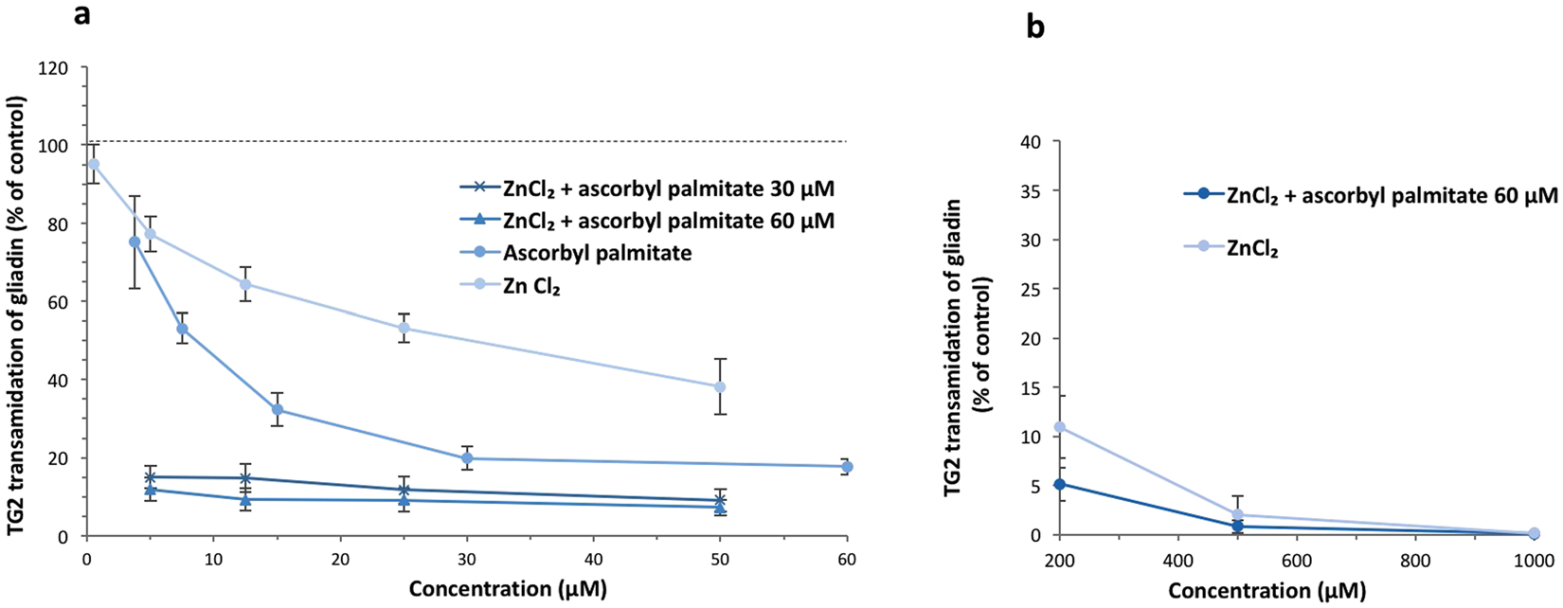
Dose-response experiments (5–1000 µM) of TG2-mediated transamidation of biotin and gliadin in the presence of ZnCl_2_ and fixed concentrations of ascorbyl palmitate. (**a**) The effect of ZnCl_2_ (5–50 µM) on TG2 activity in the presence of ascorbyl palmitate at 30 and 60 µM. (**b**) 500 µM of ZnCl_2_ in combination with ascorbyl palmitate at 60 µM was needed to completely inhibit TG2 activity. Data are presented as means ± SD (n = 3).

We introduced a Zn salt, ZnCl_2_, to completely suppress the TG2 activity in the presence of ascorbyl palmitate. Ascorbyl palmitate and ZnCl_2_ dose-response curves show a decrease in TG2 transamidation activity by the two ligands separately, and in combination, a synergistic effect was observed (Fig. 7a). Using two different inhibitory ligands allows the use of lower concentrations, which may prevent reaching the limit for the daily acceptable intake for each ligand. Dose-response experiments with ascorbyl palmitate (60 µM) at different concentrations of added ZnCl_2_ (5–1000 µM) resulted in complete inhibition (99%±1%) in the presence of 500 µM of ZnCl_2_, Fig. 7b.

## Discussion

Ascorbyl palmitate is a GRAS compound (Generally Recognized As Safe) approved by the FDA (U.S. Food and Drug Administration) as a food additive. It is frequently used as an antioxidant or as a fat-soluble form of vitamin C. The European analogue of FDA, EFSA (The European Food Safety Authority) has in line with the American authorities also concluded that there is no safety concern regarding ascorbyl palmitate (E304i) as a food additive. Back in 1973, the ADI (acceptable daily intake) of ascorbyl palmitate was estimated to 1.25 mg/kg bw/day. In the EFSA report, ascorbyl palmitate is assumed to be fully hydrolysed into ascorbate and palmitate in gastrointestinal conditions (based on unpublished data, Beck *et al*.)^10^. Ascorbic acid is frequently used as flour treatment, because of its reducing properties it forms chains in the protein network during bread making and thereby creates a strong and elastic dough. Introducing a fatty acid ester of ascorbic acid, already used as food additive, into special products for celiacs should therefore not cause a problem in terms of producer/consumer acceptance as long as proven safe. Then further studies must address the stability of ascorbyl palmitate, if it is hydrolysed already during baking or if the hydrolysis require the harsh environment of the stomach (pH 1–2). Another question is how the interaction between gliadin and ascorbyl palmitate affects the proteolytic stability of the complex and the separate ligands in the gastrointestinal tract and of course how the digestion of those affects TG2 binding to gliadin. In our studies, we conducted *in vitro* dose response experiments to confirm that TG2 binding could be estimated to decrease at increasing concentrations of ascorbyl palmitate and that saturation of gliadin occurs. If we would attempt to translate the results into the human situation, assuming that the gluten fraction would contain only α type gliadin, which contains most TG2 binding motives (nine of them), there would be a requirement of 69 mg ascorbyl palmitate per slice of wheat bread (50 g wheat flour) to completely cover all binding motives. Assuming that ascorbyl palmitate is not hydrolysed into ascorbic acid and palmitate, and that the FDA approved RDA from 1973 is still valid, it would mean that an individual of 55 kg body weight could eat 1 slice of wheat bread per day to stay within the daily limit of ascorbyl palmitate intake. A better alternative would be to focus on fortifying rye or barley products with ascorbyl palmitate, in which the content of prolamins is significantly less. So clearly, wheat flour may not be the best candidate for ascorbyl palmitate fortification on its own, therefore we conducted experiments with the TG2 inhibitor Zn as a potential way to lower the deamidation/transamidation activity of TG2 in response to gliadin. We emphasize that we are aiming for decreasing the endogenous TG2 binding to gliadin in the intestines and that we are not speaking of the addition of TG2 as a processing tool.

We found a synergistic effect of decreasing the enzymatic activity (by Zn) in combination with adding the (gln-x-pro) high affinity ascorbyl palmitate at a ratio of 1:6, which would translate into 11,6 mg Zinc in a slice of ascorbyl palmitate fortified wheat bread. However, we did not explore the synergistic effect further in respect to lowering both the ascorbyl palmitate and Zinc concentration. The effective level of Zinc is depending on intestinal TG2 levels and cannot be translated into the human situation by *in vitro* experiments and must therefore be evaluated *in vivo.* The recommended daily intake (in the U.S.) for zinc is 11 mg/day for men and 8 mg/day for women^11^ whereas the intestinal absorption of zinc from the diet has been reported to be 15–60%. Dietary zinc supplements come in daily doses in the range 10–50 mg and a common supplemental dose is about 30 mg/day. An upper tolerable level for zinc has been estimated to 50 mg/day for adults and 10 mg/day for children from 4 years (E.U.). Symptoms of mildly overdosing zinc is mainly related to gastrointestinal discomfort while yet higher doses negatively affect copper balance. Zinc salts such as ZnCl_2_, Zn acetate, and Zn sulphate are approved within the E.U. as dietary zinc supplements. Further studies must establish the quantity of Zinc needed to inhibit intestinal TG2 processing of prolamin containing flours. If there is a potential risk for reaching the limits, a solution could be to use the additive in ready-made products labelled with recommended intake quantities, to reduce the risk for adverse effects. Another potential application could be to use only gliadin/ascorbyl palmitate/ZnCl_2_ as an additive to other types of (non-gluten) flour and thereby possibly improve the visco-elastic properties of gluten-free flours. Our data supports the combination of ascorbyl palmitate with Zinc chloride as a potential gluten detoxifier with the future perspective to become a gluten detoxifying additive to flour products for the making of celiac-safe products.

## Methods

### Homology model of α2-gliadin

The FASTA file of 33 amino acid α2-gliadin (33-mer) was obtained from the complete alpha-gliadin NCBI protein (290 residues, GI: 7209265). The crystallographic coordinates of several template peptides were obtained from RSBC Protein Data Bank (PDBid: 1S9V^8^, 4OZF, 4OZG, 4OZH, 4OZI^9^). Homology model of alpha-2-gliadin was built using crystal structures of selected templates employing the YASARA program^12^. When using multiple templates (i.e. 1S9V, 4OZF, 4OZG, 4OZH, 4OZI), alternative alignments were created for each available template through a stochastic approach^13^, and the models built from these. Side-chains were built in and then subjected to a combined steepest descent and simulated annealing minimization (i.e. the backbone atoms were kept fixed). Finally, an unrestrained simulated annealing minimization was run for the entire model. The final model was evaluated by calculating the percentage of conformations in favoured regions obtained from Ramachandran plots^14^ using the PROCHECK analysis^15^.

### Virtual screening of molecules docking to α2-gliadin

Virtual screening studies were performed using Schrödinger 2015-4, employing the Maestro graphical interface^16^. Ligands were selected from a database of GRAS substances available at the US Food and Drug Administration’s website. In total, a database of 108 potential α2-gliadin ligands was built and prepared in Schrödinger, taking into account all possible tautomers, generating different stereoisomers and considering different protonation states. After preparation, the final database contained 1174 potential ligands to be employed in the virtual screening campaign. The 33-mer peptide was also prepared in Maestro (protein preparation wizard). Protonation states were assigned according to the pKa values and a pH of 7. Finally, the structure was refined through a restrained minimization using the OPLS2005 molecular mechanics force field to within a rms gradient of 0.1 kcal mol^−1^ Å^−1^ ^17, 18^. The docking grid was generated by selecting a site centred on residues 9–12 (PQLP) of the 33-mer peptide. Finally, a box able to accommodate ligands with length up to 20 Å was built. Docking was performed using the extra precision Grid-Based Ligand Docking with Energetics (Glide XP) algorithm, which docks ligands flexibly. In XP docking a better correlation between excellent poses and good scores is provided, weeding out false positives^19^. The free energy of binding was calculated using Prime/MM-GB/SA approach^20^. The docked poses were energy minimized using Prime, and the energies of the complexes were calculated using the OPLS2005 force field and generalized Born/surface area (GB/SA) continuum solvation model^21, 22^.

### Evaluation of gliadin and modelled ligands by transamidation experiments

The transamidation assay was performed as previously^23^ with modifications, but originally developed by Skovbjerg *et al*.^24^. Gliadin from wheat (G3375; Sigma Aldrich, St. Louis, MO, USA) was manually ground, dissolved in ethanol (70%) and sonicated (3 × 5 min; Elmasonic S 15; Elma Schmidbauer GmbH, Singen, Germany). 96-well plates (black Microfluor 2; Thermo Fisher Scientific, Waltham, MA, USA) were coated with gliadin (0.125 µg/well; 1 h) and washed 2 times (TBS-T 0.05%) prior to blocking (1 h; coating stabilizer and blocking buffer, art. no. C9483; Sigma Aldrich, St. Louis, MO, USA). The test molecules (Sigma Aldrich, St. Louis, MO, USA) were dissolved and diluted in TBS (5 mM; BioRad, Hercules, CA, USA) except for ergocalciferol, sodium stearate, and ascorbyl palmitate, which were first dissolved in ethanol (95%) and then diluted in TBS (final concentration 5 mM TBS and 1% ethanol). The test molecules and appropriate controls (at reported concentrations) were added to the blocked gliadin-plate. A recombinant human transglutaminase 2 (TG2; art. no. T022; Zedira GmbH, Darmstadt, Germany) was used to incorporate 5-(biotinamido)pentylamine (Thermo Fisher Scientific, Waltham, MA, USA) to the plate-bound gliadin (1 h; 37 °C). After washing (TBS-T 0.05%; 3 times), the plate was incubated with Eu-labeled streptavidin (Perkin Elmer, Waltham, MA, USA) intended for binding to gliadin-transamidated biotin. After another washing step (3 times) to remove unbound Eu-streptavidin, enhancement solution (Delfia, Perkin Elmer) was added and the fluorescence emission was measured by time-resolved fluorescence spectroscopy (345 nm excitation, 617 nm emission; Safire2; Tecan Group Ltd., Männedorf, Switzerland). The data analysis was done in Microsoft Excel.

### Deamidation experiments

The Amplex® ELISA development kit (Invitrogen, Paisley, UK) was used. Microplates were coated with gliadin (0.02 mg/ml in 70% EtOH and 10 mM DTT) and incubated in room temperature (2 h) and then washed (3 × TrisHCl-tween). The gliadin coated wells were treated with ascorbyl palmitate (60 µM), ZnCl_2_ (500 µM), or Tris-HCl and incubated for 1 h in room temperature. TG2/CaCl_2_ solution was added to the wells at 1 U/ml (final concentration) and incubated for 2 h in 37 °C and 50 rpm. After washing, the wells were blocked in Sigma’s Coating stabilizer and blocking buffer (SigmaAldrich; Schnelldorf, Germany) and incubated overnight in +4 °C and 30 rpm. In the morning, the primary antibody (mouse anti-deamidated gliadin (ab36729; Abcam) was added to the wells at 1 µg/ml and incubated in room temperature (2 h; 40 rpm). Wells were washed (3 × TrisHcl-tween 0.05%) before the incubation with the secondary antibody; goat anti-mouse IgG conjugated to HRP (A4416 Sigma; 1 µg/ml; 2 h; 40 rpm; room temperature). After the final washing step (3 × TrisHcl-tween 0.05%), the wells were added the reaction mixture containing the Amplex® UltraRed reagent. The signal was detected after 30 minutes with a microplate reader (excitation wavelength 530 nm/emission wavelength 590 nm; Tecan GmbH, Salzburg, Austria).

### Statistical analysis

The results of the experimental analysis are based on three separate runs for each sample, resulting in a total of 10–12 measurements of each sample and 42–70 measurements for the controls. The signals of the samples were first related to its respective control for each run and then these values were combined for significance testing. Two-tailed Welch’s t-tests were used for significance testing and differences were considered to be significant at p < 0.05. All statistical analyses were done in Microsoft Excel 2013.
